# Serum metabolites as early detection markers of non-muscle invasive bladder cancer in Chinese patients

**DOI:** 10.3389/fonc.2023.1061083

**Published:** 2023-03-03

**Authors:** Yi Zhao, Wei Sun, Zhigang Ji, Xiaoyan Liu, Yi Qiao

**Affiliations:** ^1^ Department of Urology, Peking Union Medical College Hospital, Chinese Academy of Science, Beijing, China; ^2^ School of Basic Medicine, Institute of Basic Medical Sciences, Peking Union Medical College, Chinese Academy of Medical Sciences, Beijing, China

**Keywords:** serum metabolites, NMIBC, LC-HRMS, bladder cancer, biomarkers

## Abstract

**Background:**

Biomarkers of different stages and grades of bladder cancer (BC) are important in clinical work. The objective of our study was to investigate new biomarkers of early-stage BC with liquid chromatography-high resolution mass spectrometry (LC-HRMS) using serum samples.

**Methods:**

A total of 215 cases were included in our study, including 109 healthy adults as the control group and 106 non-muscle invasive bladder cancer (NMIBC) patients as the NMIBC group. Serum samples were collected from BC patients in the early stage, called NMIBC, and healthy people before surgery. We used LC-HRMS to distinguish the NMIBC group from the control group and the low-grade NMIBC group from the control group.

**Results:**

An apparent difference between the NMIBC group and the control group was visualized by unsupervised principal component analysis (PCA). Metabolite panels for 16-hydroxy-10-oxohexadecanoic acid, PGF2a ethanolamide, sulfoglycolithocholate, and threoninyl-alanine were used to distinguish the two groups. The area under the curve (AUC) of the panels was 0.985, and the sensitivity and specificity were 98.63% and 98.59%, respectively. To distinguish the low-grade NMIBC group from the control group, serum metabolic profiling differences between the low-grade NMIBC group and control group samples were also analyzed. Metabolite panels of L-octanoylcarnitine, PGF2a ethanolamide, and threoninyl-alanine showed good discrimination performance. The AUC of the panels was 0.999, and the sensitivity and specificity were 97.8% and 100%, respectively.

**Conclusion:**

Metabolomics analysis of serum samples can distinguish the NMIBC group from the control group, particularly the early-stage low-grade NMIBC group.

## Introduction

The incidence of bladder cancer (Bca) has increased in recent decades. Bca is still a worldwide problem in people over the age of 50, and the incidence differs among races, with a lower morbidity but a poor survival rate in African Americans (1). The mortality rates for men and women are similar, and the development risk is four times higher in men than in women (2). The risk factors for Bca include age, race, sex, body mass index (BMI), smoking, pathogen infections, and socioeconomic status. Further studies have suggested that smoking is associated with tumor grade and stage in bladder cancer (3). In fact, all risk factors can be reflected in metabolic profiles determined by untargeted metabolomics analysis (4, 5), which is why collecting epidemiological data is important.

The current diagnosis of Bca includes cystoscopy, urinary cytology, computed tomography (CT), ultrasound (US), and magnetic resonance imaging (MRI). Bladder tumor antigen (BTA) and nuclear matrix protein 22 (NMP-22) are traditional biomarkers that have low sensitivity and specificity. Urinary cytology has low sensitivity, and cystoscopy is expensive and invasive. Tumors are classified into two different types: non-muscle invasive bladder cancer (NMIBC) and muscle-invasive bladder cancer (MIBC). Transurethral bladder tumor resection (TURBT) is the main method for treating Bca in its early stages. However, the sensitivity and specificity of cystoscopy and urinary cytology are low, and radiological examination has difficulty detecting some types of NMIBC in the early stage, for instance, papillary tumors limited to the mucosa and carcinoma *in situ* (CIS). Because of these problems, biomarkers and metabolic products with high sensitivity and specificity are necessary.

As individual risk prediction markers, metabolites have been identified with disease signatures (6). In earlier studies, Lin et al. developed a comprehensive LC−MS-based method to determine the global serum profiles of bladder cancer and kidney cancer. The results showed a high sensitivity and specificity of serum metabolites, such as eicosatrienol, docosatrienol, azaprostanoic acid, and 14’-apo-beta-carotenal, for bladder cancer (7). Liu et al. (8) also determined the global plasma profiles of 64 patients with bladder cancer, 74 patients with renal cell carcinoma, and 141 healthy people in the control group. The LC−MS-based method was used, and the results confirmed 9,10,13-TriHOME, 12,13-DHOME, and linolenelaidic acid glycocholic acid as the best prediction biomarkers for bladder cancer. Other metabolites, such as hypoxanthine, tryptophan, malonate, lactate, dimethylamine, glutamine, histidine, and valine, were reported to differentiate Bca patients from healthy people (1). However, the sample sizes in all the previous studies were small, and not all grades and stages of bladder cancer were detected.

In this study, we further studied serum metabolic profiling in the diagnosis of NMIBC. Furthermore, we discovered biomarker panels that could distinguish between NMIBC patients and controls, low-grade NMIBC patients and controls, and high-grade NMIBC patients and controls.

## Methods

### Sample collection and preparation

All patients and their families provided informed consent before the study. According to the 2004 classification criteria of the World Health Organization/International Society of Urological Pathology, papillary urothelial proliferation of low-malignant potential (PUNLMP), low-grade papillary carcinoma, and high-grade papillary carcinoma NMIBC patients were collected. The serum samples were all collected before the TURBT operation and centrifuged within 6 hours, and the supernatants were stored at −80°C after isolation and aliquoting. The process was as follows: 200 µl of serum was mixed with 200 µl of acetonitrile and vortexed for 30 s. Then, the samples were centrifuged at 14,000×*g* for 10 min. After that, the supernatant was dried and stored. Before transferring the samples to an autosampler, 10 kDa molecular weight cut-off ultracentrifugation filters (Millipore Amicon Ultra, MA) were used for small-protein depletion. The dried powder that remained was reconstituted in 200 µl of 2% acetonitrile. This research was approved by the Institutional Review Board of the Institute of Basic Medical Sciences, Chinese Academy of Medical Sciences.

### Analysis steps

A Waters ACQUITY H-class LC system coupled with an LTQ-Orbitrap mass spectrometer (Thermo Fisher Scientific, MA, USA) was used. A Waters HSS C18 column (3.0 × 100 mm, 1.7 µm) was used to perform metabolite separation at a flow rate of 0.5 ml/min using a 17-minute gradient. The gradient was set at 1–3 min, 2%–55% solvent B; 0–1 min, 2% solvent B; 3–8 min, 55%–100% solvent B; 8–12 min, 100% solvent B; 12–12.1 min, 100–2% solvent B; and 12.1–17 min, 2% solvent B. The mobile phases were 0.1% formic acid in H2O (A) and acetonitrile (B). The column temperature was 50°C. The mass scan ranged from 100 to 1,000 m/z. The MS1 analysis resolution was set at 60 K, and the MS2 resolution was 15 K. The automatic gain control was set as 1 × 106 with a maximum injection time (IT) of 100 ms. The MS2 automatic gain control was set as 5 × 105, and the maximum IT was 50 ms. Metabolites were dissociated by higher-energy collisional dissociation (HCD) fragmentation mode with an optimal collision energy of 20, 40, 60, or 80.

### Data normalization and processing

Raw data files were processed by Progenesis QI (Version 2.0, Nonlinear Dynamics) software. The mass list data file exported from QI was further processed by MetaboAnalyst 5.0 (http://www.metaboanalyst.ca), including missing value estimation, log2 transformation, and Pareto scaling. Variables missing in 50% or greater of all samples were removed from further statistical analysis, and the other missing values were filled using the KNN method. Pattern recognition analysis (principal component analysis, PCA; orthogonal partial least squares discriminant analysis, OPLS-DA) was performed using SIMCA 14.0 (Umetrics, Sweden) software. The variable importance for projection (VIP) value obtained from OPLS-DA was used for differential metabolite selection. Nonparametric tests (Wilcoxon rank sum test) were used to evaluate the significance of variables. False discovery rate (FDR) correction (Benjamini method) was used to estimate the chance of false positives and correct for multiple hypothesis testing. Differential metabolites were selected according to the following criteria: (1) VIP value >1; (2) adjusted p-value <0.05. A ROC curve was constructed based on differential metabolites using a logistic regression algorithm.

## Results

### General information

The sample size was calculated according to plasma proteome individual variation and the fold change cut-off used in the present differential analysis. The median interindividual variation for quantitative proteins was 0.6 in plasma. The minimum sample size at 5% FDR is shown in the following tables. When the fold change was 1.5, the average minimum sample size was approximately 48 (**Table S1**).

In the present study, 215 cases were enrolled, including 109 healthy adults as the control group and 106 NMIBC patients as the Bca group. In the NMIBC group, 72 cases were in the discovery set, with 70 cases being single tumors and two cases being multiple tumors, and 34 were in the external validation set, with all of them being single tumors. Most of the tumors were individual, tumor size was not an influence factor, and the invasion of the bladder muscle was the key to the TNM stage and pathological type. So, we did not analyze the number of tumors or their size. All our NMIBC include the stages of Tis, Ta, and T1. BUS, CT, or cystoscope could only find T1 tumors, while Tis and Ta may have to depend on other more advanced methods, such as fluorescent cystoscopes, to detect them. The pathology of our patients was all adenomas. At last, we compared the NMIBC (Tis, Ta, or T1) group and the control group, as well as the low- and high-grade NMIBC group and the control group. So, TNM stage and pathological type did not influence the results of the study. The detailed general information is listed in **Table 1**.

**Table 1:** General information of NMIBC vs Control group and Low-grade NMIBC vs Control group. .

	NMIBC vs Control				Low-grade NMIBC vs Control	
	NMIBC		Control		Low-grade NMIBC	Control
	Discovery set	External validation set	Discovery set	External validation set		
Cases	72	34	73	36	46	46
Ages	64.16±12.38	63.58±11.86	57.6±10.87	58.02±10.63	59.8±13.7	57.7±10.8
Sex (male/female)	53/19	26/8	46/27	22/14	32/13	32/13
Tumor numbers (average)	1.03	1	0	0	1	0
Tumor sizes	1.11±0.38	1.03±0.22	–	–	1.4±0.98	–

**Figure 1:**
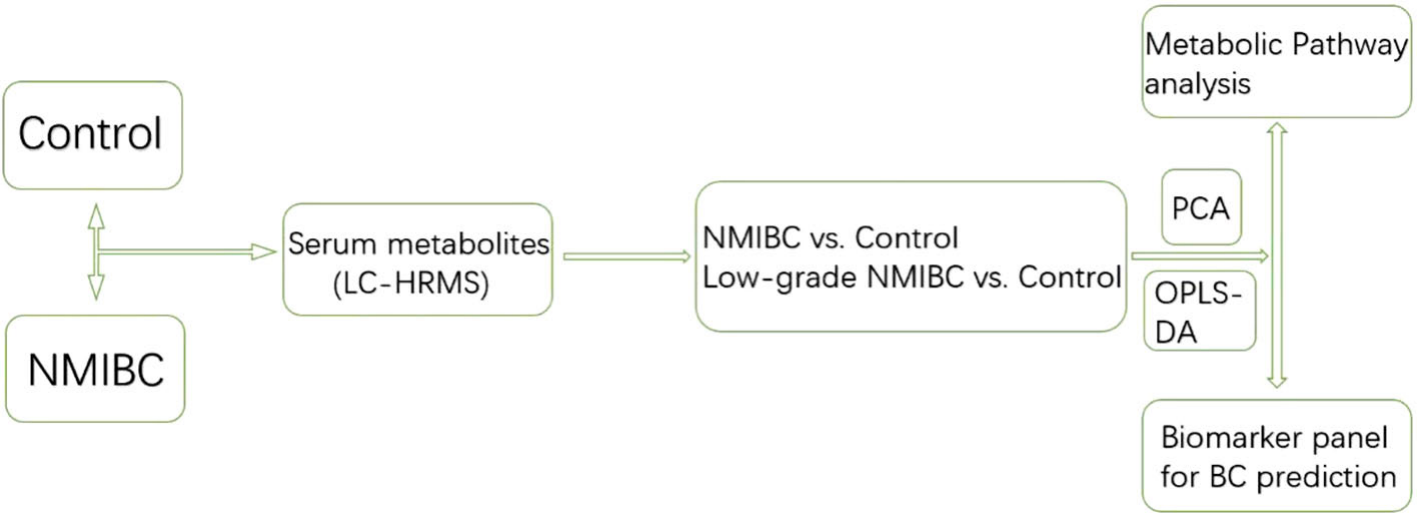
The workflow of the analysis.

Serum metabolic profiling differences between the control group and NMIBC samples were analysed. Potential biomarkers were discovered to distinguish NMIBC. Additionally, to perform early detection of Bca, serum metabolic profiling differences between the control group and early-stage low-grade cases were also analyzed. The workflow is shown below (**Figure 1**).

### Quality control

This cohort of samples was analyzed in one center, and quality control (QC) was important to limit bias. Almost every 5 days, all samples are analyzed. The QC samples were prepared using a total of 80 representative samples mixed in equal aliquots, and they were injected every 10 samples. PCA showed that QC samples clustered slightly. The QC results showed that biological variations are the key to distinguishing group differences (**Figure S1**).

### NMIBC group *vs*. control group

**Figure 2:**
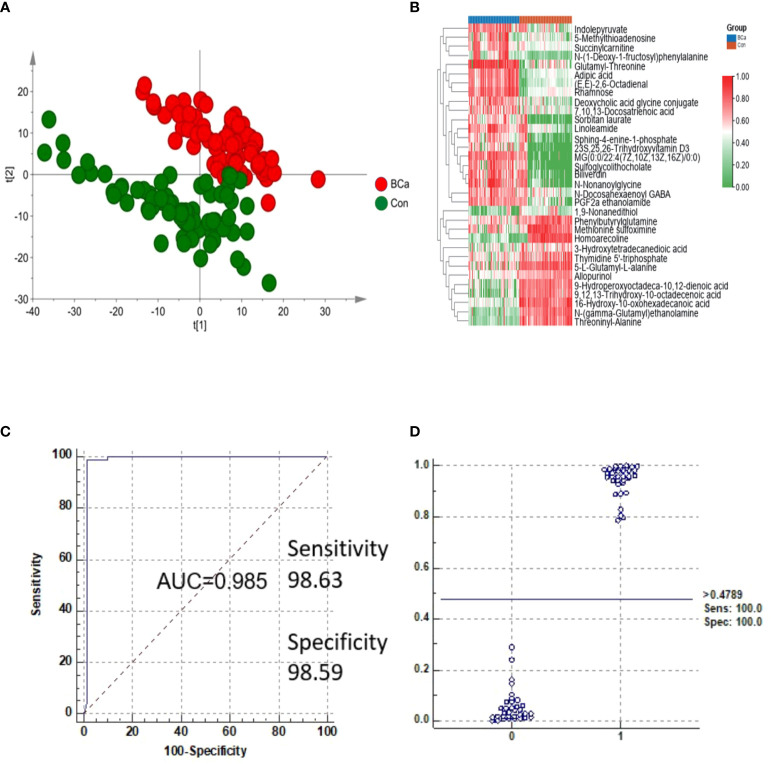
Analysis of metabolites in the NMIBC group *vs*. the control group. **(A)** Score plot of PCA of the NMIBC group and control group. **(B)** Relative intensity of differential metabolites in the NMIBC group and control group. **(C)** ROC plot of the NMIBC group and control group based on the discrimination degree. **(D)** External prediction of NMIBC by metabolite panels of 16-hydroxy-10-oxohexadecanoic acid, PGF2a ethanolamide, sulfoglycolithocholate, and threoninyl-alanine.

Multistatistical analysis was used to distinguish the NMIBC group from the control group. Metabolic profiling differences between the control group and NMIBC group subjects were visualized by unsupervised PCA (**Figure 2A**), and a supervised OPLS-DA model was established for better separation. Differential metabolites were selected by the following criteria: value importance in the projection (VIP) >1, adjusted p-value <0.05. Finally, 33 metabolites were detected, with 20 increased and 13 decreased (**Figure 2B**). We further performed pathway enrichment analyses. Pathways including tyrosine metabolism, porphyrin metabolism, fructose metabolism, and phosphatidylinositol phosphate metabolism varied between the NMIBC and control groups.

To detect potential markers for distinguishing NMIBC patients from normal controls, ROC curves and multivariate ROC curves were used to evaluate the diagnostic accuracy of the differential metabolites for NMIBC. The area under the curve (AUC) values of the 33 metabolites were greater than 0.8, which indicates their potential value in clinical diagnosis. We selected the potential biomarker panel based on its biological significance and used K-means (KM) clustering to detect features with similar behavior to help reduce the redundancy in biomarkers (i.e., features in the same cluster behave more similarly). K-means (KM) clustering was performed by MetaboAnalyst 5.0 (“Biomarker discovery” module). Finally, metabolites of 16-hydroxy-10-oxohexadecanoic acid, PGF2a ethanolamide, sulfoglycolithocholate, and threoninyl-alanine were used to construct a predictive model for NMIBC (**Table S2**). The AUC of the metabolite panels was 0.985, and the sensitivity and specificity were 98.63% and 98.59%, respectively (**Figure 2C**). Biomarkers were also validated in another batch of samples consisting of 34 NMIBC samples and 36 control samples, and the results showed that sensitivity and specificity were 100% and 100%, respectively (**Figure 2D**).

### Analysis of low-grade NMIBC group *vs*. the control group

**Figure 3:**
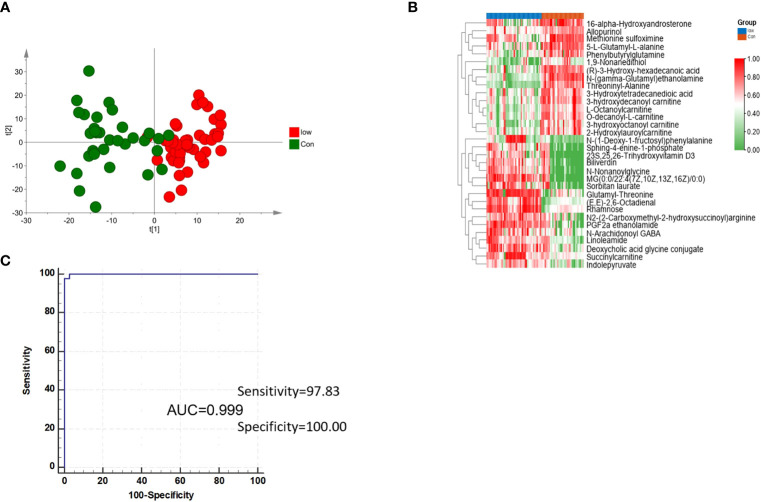
Analysis of metabolites in the low-grade NMIBC group *vs*. the control group. **(A)** Score plot of the OPLS-DA model analysis of the low-grade NMIBC group and the control group. **(B)** Relative intensity of differential metabolites in the low-grade NMIBC group and the control group by metabolite panels of L-octanoylcarnitine, PGF2a ethanolamide, and threoninyl-alanine. **(C)** ROC plot of the low-grade NMIBC group and control group based on the discrimination degree.

Exploring potential biomarkers for the early differential diagnosis of the low-grade NMIBC group and the healthy group is very important in clinical work and treatment. PCA and the OPLS-DA model were used again to discriminate between the control group and the low-grade NMIBC group (**Figure 3A**). VIP >1 and adjusted p-value <0.05 were used to select differential metabolites. Thirty-two metabolites, 17 increased and 15 decreased, were ultimately identified (**Figure 3B**).

Then, ROC curves and multivariate ROC curve analysis were used to detect markers that distinguished the low-grade NMIBC from the control group, and a total of 32 significantly differential metabolites were found. Further K-means clustering was used to select potential biomarkers. Using a similar strategy for biomarker selection, three metabolite panels were determined to perform the best at distinguishing elements between the control group and the low-grade NMIBC group, including L-octanoylcarnitine, PGF2a ethanolamide, and threoninyl-alanine (**Table S3**). The sensitivity and specificity were 97.8% and 100%, respectively (**Figure 3C**).

## Discussion

With the development of diverse treatment methods, early detection or diagnosis is increasingly important, especially in NMIBC patients. In the past decade, serum or urine metabolomics have been promising methods for the early detection of Bca. Urinary metabolites, which are produced by several methods, including mitochondrial oxidative phosphorylation, glycolysis, cholesterol metabolic pathways, and fatty acid metabolism (9), have been studied at relatively high levels in recent years. Felice et al. (10) detected that liquid biopsy, which was detected in urine, could overcome the limitations of the sensitivity of cytology and played an important role in the prognosis and surveillance of bladder cancer.

Our study showed that four metabolite panels were found to distinguish NMIBC patients from healthy people in serum, and three metabolite panels were found to distinguish low-grade NMIBC patients from healthy patients. In our literature review, we identified metabolic biomarkers mostly from urine samples, and serum samples were less common. In our experience, urine samples were collected by patients themselves, which were easily polluted, and serum samples were collected by nurses, which were non-polluted. Because of the characteristics of small sizes and non-polluted serum samples, we chose serum as our ideal sample.

PGF2a ethanolamide and threoninyl-alanine are both sensitive in NMIBC patients, especially low-grade NMIBC patients. PGF2a ethanolamide was first reported as a treatment in open-angle glaucoma therapy, which was proved to be associated with high-density lipoprotein (HDL) serum levels (11). To our knowledge, this is the first time PGF2 has been detected as a biomarker for bladder tumors. Fortunately, PGF2a ethanolamide is both positive in the NMIBC *vs*. control group and in the low-grade NMIBC *vs*. control group; however, the result was negative in the high-grade NMIBC group. In the previous study, we suggested that PGF2a ethanolamide was sensitive to bladder cancer in early and low-grade stages. More studies were necessary, and we will make a further confirmation to detect whether PGF2 had a relationship with NMIBC and lipid, especially HDL level.

Yasushi et al. (12) explored the alanine (Ala) to valine (Val) polymorphism in exon 2 and the isoleucine to threonine polymorphism at codon 56 of the manganese superoxide dismutase gene (MnSOD) to further define the risk factors for bladder cancer with 213 patients and 209 normal controls. The results confirmed that the GPX1 genotype may further affect the status of bladder cancer, and the increased risk may be modified by the Ala-9Val MnSOD polymorphism. While this is the first time that threoninyl-alanine has been reported in NMIBC and low-grade bladder cancer, and even in all the tumors, the sensitivity and specialty were high. Threoninyl-alanine in MIBC patients should be clarified in the future.

Our results further confirmed that 16-hydroxy-10-oxohexadecanoic acid was only sensitive to low-grade bladder cancer, and the function of 16-hydroxy-10-oxohexadecanoic acid was limited in the detection of bladder cancer at an early stage. Liu et al. (8) reported that 16-hydroxy-10-oxohexadecanoic acid, which was downregulated in Bca and kidney cancer (KC) groups compared with the control group, showed the best predictive ability with a ROC area of 1 (AUC = 0.994) for the testing dataset. Our results were similar: the ROC area in the discovery set was 0.988, and the ROC area in the external validation set was 0.997. However, while Liu et al. detected bladder cancer with LC–MS analysis, they did not make a further study of bladder cancer at different stages.

L-Octanoylcarnitine was reported and proven by Zoni et al. (13) showed a relationship between the metabolite profile of preoperative prostate cancer (PCa) patients and the risk of PCa progression. Zoni et al. detected that L-octanoylcarnitine was positively associated with suberic acid and correlated with the risk of PSA progression (p = 0.032, log-rank test). L-Octanoylcarnitine and sulfoglycolithocholate are reported for the first time in bladder cancer in our study.

We also determined that distinguishing between the high-grade NMIBC group and the low-grade NMIBC group is very important in clinical work and treatment. PCA was also performed, and the results showed that there were no significant differences between the high-grade NMIBC group and the low-grade NMIBC group. The following reasons may explain the results (1): the sample size was small, which may produce bias, or (2) the tumor cytological structure was too like distinguish them from each other in the two groups. There are also some limitations in our study: (1) In our previous study, urinary metabolites had been analyzed in NMIBC patients, and dopamine 4-sulfate, MG00/1846Z,9Z,12Z,15Z/00, aspartyl-histidine, and tyrosyl-methionine were found to distinguish NMIBC patients, and 3-hydroxy-cis-5-tetradecenoylcarnitine, 6-ketoestriol, beta-cortolone, tetrahydrocorticosterone, and heptylmalonic acid were found to distinguish low-grade NMIBC patients (14). Further study is going to be done by analyzing serum and urine metabolites together to increase the diagnostic sensitivity and specificity of bladder cancer. (2) More studies of markers as having a prognostic role were detected in our literature review (15); however, in our study we did not analyze the markers as having a prognostic role. We will collect more information about bladder cancer prognostic factors and detect an important role.

## Conclusion

Metabolomics analysis of serum samples was able to distinguish the NMIBC group from the control group and the low-grade NMIBC group from the control group. Further studies are required to find more biomarkers based on larger samples and multiple centers.

## Data availability statement

The datasets used and/or analysed during the current study are available from the corresponding author on reasonable request.

## Ethics statement

This research was approved by the Institutional Review Board of the Institute of Basic Medical Sciences, Chinese Academy of Medical Sciences. All patients and their families were provided informed consent before the study.

## Author contributions

YZ: Manuscript writing/editing, Data collection or management. WS: Protocol/project development. ZJ: Protocol/project development. XL: Data analysis. YQ: Data collection or management. All authors contributed to the article and approved the submitted version.
