# Supplementary material for: Serum metabolites as early detection markers of non-muscle invasive bladder cancer in Chinese patients
Source: Front Oncol. 2023 Mar 3;13:1061083. doi: 10.3389/fonc.2023.1061083 (PMC10020364; doi:10.3389/fonc.2023.1061083)
Supplement: Supplementary file 1 [file Table_1.docx]

**Table S1. The calculation of sample size with fold change cutoff used in different analysis.**

| fold change | power | a | π | FDR | n |
| --- | --- | --- | --- | --- | --- |
| 1.5 | 0.8 | 0.001 | 0.05 | 2.32% | 52 |
| 1.5 | 0.8 | 0.005 | 0.1 | 5.33% | 41 |
| 1.5 | 0.8 | 0.01 | 0.2 | 4.76% | 36 |
| 1.5 | 0.9 | 0.001 | 0.05 | 2.06% | 63 |
| 1.5 | 0.9 | 0.005 | 0.1 | 4.76% | 51 |
| 1.5 | 0.9 | 0.01 | 0.2 | 4.26% | 45 |
