# Supplementary material for: Serum metabolites as early detection markers of non-muscle invasive bladder cancer in Chinese patients
Source: Front Oncol. 2023 Mar 3;13:1061083. doi: 10.3389/fonc.2023.1061083 (PMC10020364; doi:10.3389/fonc.2023.1061083)
Supplement: Supplementary file 2 [file Table_2.docx]

| **Table S2 Differential metabolites between Control and NMIBC** | | | |  |
| --- | --- | --- | --- | --- |
| Description | VIP | P Adjusted | ROC | K-means cluster |
| 16-Hydroxy-10-oxohexadecanoic acid | 2.83243 | 2.06E-21 | 0.98823 | 1 |
| Threoninyl-Alanine | 2.57056 | 9.65E-21 | 0.97646 | 1 |
| N-(gamma-Glutamyl)ethanolamine | 3.37471 | 4.39E-20 | 0.96817 | 1 |
| 9,12,13-Trihydroxy-10-octadecenoic acid | 3.17682 | 6.89E-20 | 0.96489 | 1 |
| 9-Hydroperoxyoctadeca-10,12-dienoic acid | 2.99282 | 7.25E-20 | 0.96489 | 1 |
| 5-L-Glutamyl-L-alanine | 2.34943 | 1.24E-16 | 0.92205 | 1 |
| Allopurinol | 1.70685 | 2.19E-16 | 0.91858 | 1 |
| Homoarecoline | 2.51313 | 1.82E-14 | 0.8908 | 1 |
| Methionine sulfoximine | 2.57618 | 1.08E-12 | 0.86321 | 1 |
| Phenylbutyrylglutamine | 1.90769 | 1.08E-12 | 0.86321 | 1 |
| 1,9-Nonanedithiol | 2.55346 | 2.15E-09 | 0.80677 | 1 |
| Thymidine 5'-triphosphate | 1.44262 | 4.51E-08 | 0.78217 | 1 |
| 3-Hydroxytetradecanedioic acid | 1.58336 | 7.29E-07 | 0.75728 | 1 |
| MG(0:0/22:4(7Z,10Z,13Z,16Z)/0:0) | 5.83578 | 4.1343E-19 | 0.94424 | 2 |
| PGF2a ethanolamide | 3.05172 | 9.74E-16 | 0.90855 | 2 |
| Sulfoglycolithocholate | 4.56875 | 4.60E-16 | 0.90064 | 2 |
| Biliverdin | 3.69188 | 9.87E-15 | 0.89003 | 2 |
| N-Nonanoylglycine | 3.45376 | 1.63E-14 | 0.8879 | 2 |
| Sorbitan laurate | 3.12141 | 5.85E-14 | 0.87681 | 2 |
| Deoxycholic acid glycine conjugate | 2.47898 | 2.1728E-12 | 0.85838 | 2 |
| Sphing-4-enine-1-phosphate | 3.29469 | 2.138E-11 | 0.8389 | 2 |
| Linoleamide | 1.96492 | 4.02E-11 | 0.83774 | 2 |
| 23S,25,26-Trihydroxyvitamin D3 | 2.35151 | 2.34E-11 | 0.82964 | 2 |
| N-Docosahexaenoyl GABA | 1.78782 | 2.34E-10 | 0.82443 | 2 |
| 7,10,13-Docosatrienoic acid | 2.19145 | 5.95E-08 | 0.77986 | 2 |
| (E,E)-2,6-Octadienal | 3.27412 | 5.0004E-21 | 0.98071 | 3 |
| Adipic acid | 3.22137 | 5.00E-21 | 0.98109 | 3 |
| Rhamnose | 3.56025 | 8.4735E-21 | 0.97743 | 3 |
| Glutamyl-Threonine | 2.75526 | 1.77E-18 | 0.94665 | 4 |
| N-(1-Deoxy-1-fructosyl)phenylalanine | 2.33876 | 1.03E-13 | 0.87922 | 4 |
| Succinylcarnitine | 1.91105 | 4.8049E-10 | 0.81883 | 4 |
| Indolepyruvate | 1.29201 | 9.57E-09 | 0.7951 | 4 |
| 5-Methylthioadenosine | 1.1863 | 1.42E-04 | 0.70172 | 4 |
