# Supplementary material for: Serum metabolites as early detection markers of non-muscle invasive bladder cancer in Chinese patients
Source: Front Oncol. 2023 Mar 3;13:1061083. doi: 10.3389/fonc.2023.1061083 (PMC10020364; doi:10.3389/fonc.2023.1061083)
Supplement: Supplementary file 3 [file Table_3.docx]

| **Table S3 Differential metabolites between Control and low-grade NMIBC** | | | | |
| --- | --- | --- | --- | --- |
| Description | VIP | P Adjusted | ROC | K-cluster |
| Allopurinol | 1.67433 | 7.6E-10 | 0.9 | 1 |
| L-Octanoylcarnitine | 1.66698 | 8.4E-09 | 0.88075 | 1 |
| 3-hydroxyoctanoyl carnitine | 1.69586 | 2.2E-08 | 0.87205 | 1 |
| O-decanoyl-L-carnitine | 1.67066 | 3.6E-08 | 0.8677 | 1 |
| 3-hydroxydecanoyl carnitine | 1.50823 | 4.1E-08 | 0.86646 | 1 |
| Threoninyl-Alanine | 2.69642 | 1.5E-18 | 0.99627 | 2 |
| N-(gamma-Glutamyl)ethanolamine | 3.40974 | 8.6E-16 | 0.98075 | 2 |
| (E,E)-2,6-Octadienal | 3.2311 | 9.2E-11 | 0.96149 | 2 |
| Rhamnose | 3.52076 | 1.5E-13 | 0.95839 | 2 |
| Glutamyl-Threonine | 2.95363 | 1.3E-10 | 0.95714 | 2 |
| (R)-3-Hydroxy-hexadecanoic acid | 2.78561 | 1.7E-10 | 0.95466 | 2 |
| 5-L-Glutamyl-L-alanine | 2.17437 | 7.4E-09 | 0.91304 | 2 |
| Phenylbutyrylglutamine | 1.83653 | 1.9E-08 | 0.87329 | 2 |
| 1,9-Nonanedithiol | 2.88884 | 6.9E-07 | 0.85621 | 2 |
| Deoxycholic acid glycine conjugate | 2.73613 | 3.4E-07 | 0.84534 | 2 |
| N-(1-Deoxy-1-fructosyl)phenylalanine | 2.2172 | 4.7E-07 | 0.84161 | 2 |
| Succinylcarnitine | 2.16136 | 3.6E-06 | 0.81863 | 2 |
| Indolepyruvate | 1.43268 | 4.6E-06 | 0.81553 | 2 |
| 2-Hydroxylauroylcarnitine | 1.3572 | 1.7E-05 | 0.79938 | 2 |
| PGF2a ethanolamide | 3.49978 | 2.2E-09 | 0.92609 | 3 |
| MG(0:0/22:4(7Z,10Z,13Z,16Z)/0:0) | 5.63946 | 4.8E-09 | 0.91056 | 3 |
| N-Nonanoylglycine | 3.79602 | 1E-07 | 0.87826 | 3 |
| Biliverdin | 3.72869 | 3.4E-07 | 0.86335 | 3 |
| Linoleamide | 2.30536 | 9.6E-07 | 0.85217 | 3 |
| Sphing-4-enine-1-phosphate | 3.75425 | 1.1E-06 | 0.84658 | 3 |
| 16-alpha-Hydroxyandrosterone | 1.81841 | 1.1E-06 | 0.8323 | 3 |
| Methionine sulfoximine | 2.05044 | 5.1E-06 | 0.82857 | 3 |
| 3-Hydroxytetradecanedioic acid | 1.98674 | 5.5E-06 | 0.82733 | 3 |
| Sorbitan laurate | 2.6801 | 5.2E-06 | 0.8264 | 3 |
| N2-(2-Carboxymethyl-2-hydroxysuccinoyl)arginine | 1.9947 | 9E-06 | 0.8205 | 3 |
| N-Arachidonoyl GABA | 1.97457 | 1.4E-05 | 0.81491 | 3 |
| 23S,25,26-Trihydroxyvitamin D3 | 2.44879 | 1.9E-05 | 0.80186 | 3 |
