# Supplementary figures and images for: Serum metabolites as early detection markers of non-muscle invasive bladder cancer in Chinese patients
Source: Front Oncol. 2023 Mar 3;13:1061083. doi: 10.3389/fonc.2023.1061083 (PMC10020364; doi:10.3389/fonc.2023.1061083)

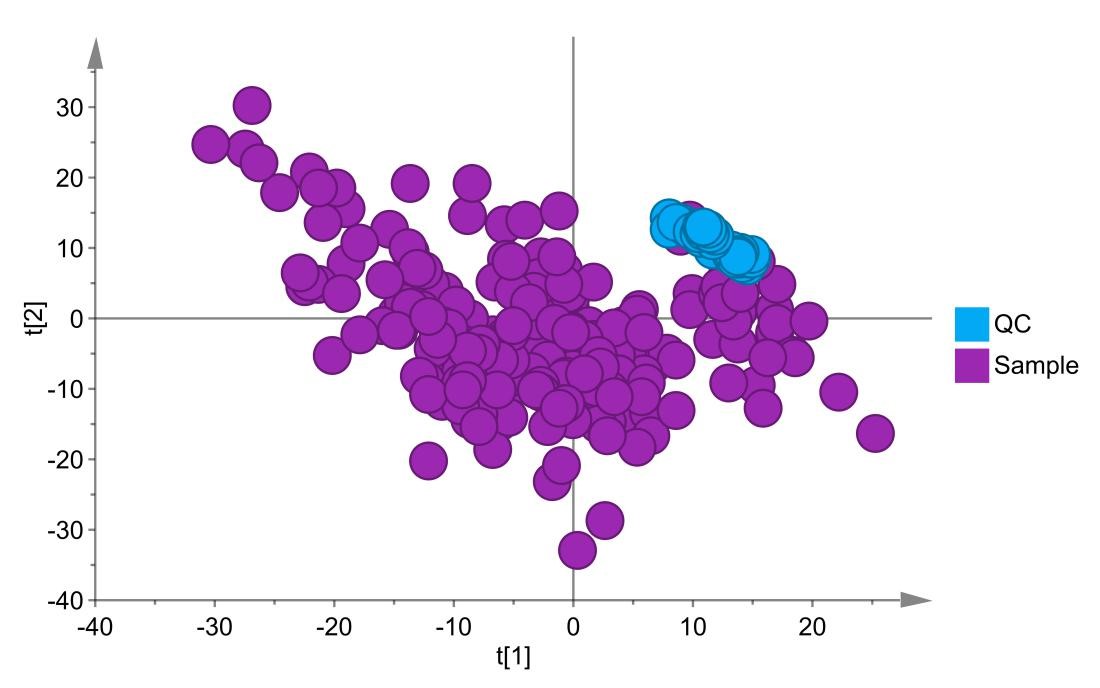

Supplement: Supplementary file 4 [file Image_1.jpeg]
